# Clustering Esports Gameplay Consumers *via* Game Experiences

**DOI:** 10.3389/fspor.2021.669999

**Published:** 2021-06-17

**Authors:** Wooyoung William Jang, Kevin K. Byon, Jennifer Pecoraro, Yosuke Tsuji

**Affiliations:** ^1^University of West Georgia, Carrollton, GA, United States; ^2^Indiana University, Bloomington, IN, United States; ^3^Rikkyo University, Tokyo, Japan

**Keywords:** esports, market segmenetation, game experience, esports gameplay, viewing esports content

## Abstract

This study focuses on “game experiences” in the context of esports gameplay consumption and aims to identify adequate consumer groups based on their esports experience including perceptions of gameplay, watching, and purchasing hardware. The purpose of this study is to identify adequate consumer groups through consumer segmentation. Based on the literature review, a matrix of esports gameplay was proposed based on high/low esports gameplay, viewing esports, and hardware enthusiasm. Four esports gameplay consumer groups are proposed (all-around gamer, conventional player, observer, recreational gamer) based on their prior esports experiences (esports gameplay, viewing esports content via media, and hardware enthusiasm). A total of 699 usable observations were initially collected by the online survey. Eventually, 508 observations were retained (127 for each group) for multivariate analysis of variance and subsequent univariate tests. The findings indicated the four esports gameplay consumer groups were empirically supported. Furthermore, this study found similarities and differences for each group based on the six antecedents of esports gameplay intention. The findings indicated hedonic motivation and price value might be considered general factors that may be applied to all esports consumers. Contrarily, the findings indicated that social influence, habit, effort expectancy, and flow might be suitable for tailored marketing strategies targeting esports consumer groups. Theoretically, the suggested esports experience will contribute to the growing body of knowledge aimed at understanding esports consumers' behavior through the consistent clustering of behavioral prior experience. Practically, the proposed esports consumers' clustering will contribute to more efficient marketing, with spending on more targeted marketing leading to effectively reaching the right people.

## Introduction

In sport management, scholars are driven to better understand sport consumption, such as the fans' motivations, constraints, negotiation, etc., from die-hard to fair-weather fans (Byon et al., 2020). As such, consumer clusters and drivers of consumer behaviors have also drawn scholars' attention in the esports context. esports continues to pique the interest of interdisciplinary, scholarly inquiries (Cranmer et al., 2021) by seeking to better understand complex consumer clusters and their consequential influences on esports consumerism in a broad sense. Among other notable research foci regarding the legitimacy of esports, Cranmer et al. (2021) noted that “a key area of research will undoubtedly evolve around the consumer market” (p. 117). In support of this assertion, a review of early scholarly esports investigations illustrates the academic propensity to explore and establish esports consumer behavior and clusters (Jang and Byon, 2020a; Reitman et al., 2020). While such seminal work has undoubtedly made significant contributions to the esports body of knowledge at large, recent literature has stressed the importance of producing cohesive and timely research regarding notable esports industry growth (Newzoo, 2019) and market trends [e.g., those caused by coronavirus disease 2019 (COVID-19)].

Among the limited research inquiries—specific to esports consumer segmentation—various scholars have sought to identify factors and moderators of esports consumption (Pizzo et al., 2018; Jang and Byon, 2020b, in press). In line with this pursuit, researchers found esports consumers are quite heterogeneous. Segmentation studies—specific to gameplay consumption—identified the esports game genre (Jang and Byon, 2020b) and gender (Jang and Byon, in press) as moderators for segmentation. In esports event spectating motivation, Pizzo et al. (2018) compared sport-simulation esports game and real-time strategy esports game segments. While useful in establishing moderators, additional research is needed to fully explore consumers' experiences with esports in a manner that cultivates distinct clusters. This disparity may be particularly problematic when seeking to adequately categorize complex esports consumers within this rapidly growing industry. Understanding consumer market segmentation is beneficial for practitioner implementation when building consumer profiles and subsequent marketing initiatives. Such work would consequently aid in bridging the gap between academia and industry practices. Additionally, without adequate segmentation, practitioners are left to operate with limited—and possibly inaccurate—data upon which to base decisions. Thus, careful consideration is needed when exploring and determining factors segmenting esports consumers.

In video gaming, research seeking to incorporate gameplay experience in the consumer segmentation process categorized videogame consumers as being a “hardcore gamer” or a “casual gamer” (Juul, 2010). While the terms “hardcore gamer” vs. “casual gamer” have been colloquially used to describe gameplay enthusiasm, the limited scholarly application of these terms requires an additional inquiry to more adequately identify the segmented clusters. To address this gap, multiple attempts have been made to expand Juul's (2010) initial segmentation (Billieux et al., 2015; Manero et al., 2016; Yee, 2019; Gamedesigning.org, 2020). While previous research has sought to segment esports consumers based on various factors and moderators, only limited research exists aiming to segment consumers according to their *game experience*. Understanding consumers' previous experiences provides scholars and practitioners alike with essential information to aid in market segmentation; previous research—specific to the influence of prior experience with technology on understanding technology acceptance and usage behavior—illustrates the extent to which evaluating game experience is essential when seeking to segment markets (Venkatesh et al., 2012). Similarly, Fishbein and Ajzen (2011) suggest Consumers' experience should be considered when seeking to understand consumption behavior. The Theory of Planned Behavior suggests consumers' previous experiences could influence their perceptions leading to behavioral intention in the context of technology and tourism (Blut and Wang, 2019; Heiny et al., 2019). Specifically, technology readiness could be influenced by an individual's situational environment and prior experience (Blut and Wang, 2019). Tourism activities could be influenced by an individual's background factor and experience (Heiny et al., 2019). As such, esports fans' game experiences might influence self-perceptions regarding the driving factors behind their gameplay consumption intention. In all, previously conducted research establishes game experience as a potentially viable scope for esports consumer segmentation. However, additional inquiries are needed to explore how game experience could produce meaningful and distinct clusters.

Often in tangent to esports segmentation research, scholars have begun developing operational models with which to better understand esports consumer behaviors. Concerning antecedents and consequences of esports gameplay intention, the esports Consumption (ESC) model (Jang and Byon, 2020a) illustrates the focal constructs of gameplay intention; gameplay intention is a critical factor in esports gameplay behavior and esports events media consumption. As esports can be defined as a competitive sport based on human–electronic device interaction, technology acceptance is inevitable to understand esports consumption. Thus, the ESC model has been grounded in the Unified Theory of Acceptance and Use of Technology 2 (Venkatesh et al., 2012). The ESC model mainly focuses on esports gameplay consumption as a primary consumption, leading to other types of esports consumption, such as media consumption. The ESC model indicates six focal antecedents of esports gameplay intention: hedonic motivation (HM), habit (HB), price value (PV), effort expectancy (EE), social influence (SO), and flow (FL). The ESC model establishes foundational tenants for understanding the driving factors behind esports gameplay intention and behavior. Applications of this model will aid researchers in more adequately segmenting esports consumers based on gameplay experience. While this model is certainly valuable regarding foundationally understanding esports consumer behaviors, additional developments are needed by incorporating boundary conditions, such as game experience.

**Figure 1:**
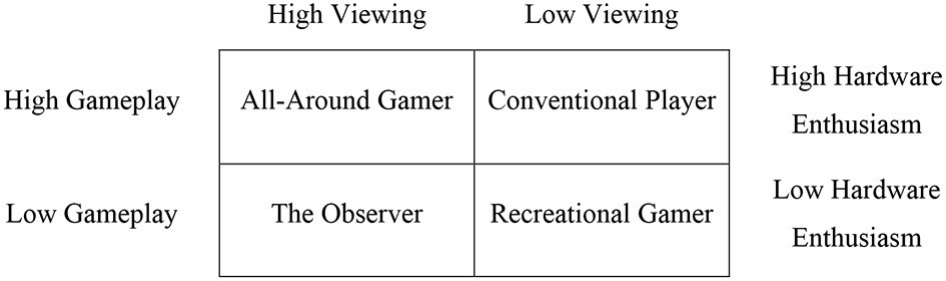
Proposed four gamer types.

The purpose of this study is to identify adequate consumer groups through consumer segmentation. Specifically, this study utilizes game experience to cultivate consumer clusters. Based on the literature review, a matrix of esports gameplay was proposed based on high/low esports gameplay, viewing esports, and hardware enthusiasm (Figure 1). According to the gaming industry phenomena, the preference for purchasing and upgrading the most recent gaming hardware is closely correlated with high gameplay consumption (Newzoo, 2019). It might be explained that the purpose of the gaming consoles and gear is to use them only for gameplay. The esports gameplay consumers may spend their money to update their gaming hardware to enjoy their favorite esports game with better visual or functional performance. Thus, hardware enthusiasm was assumed to be highly correlated with gameplay consumption as a peripheral element. For these reasons, the clusters were mainly categorized by esports gameplay and viewing esports. First, “all-around gamer” was clustered as high esports gameplay frequency, high esports content watching, and high hardware enthusiasm. All-around gamers might have balanced consumption between gameplay, viewing, and hardware enthusiasm at a high level. Second, “conventional players” are likely to play esports games frequently and maintain a high level of gaming hardware but may not be huge fans of watching esports events or streamers' esports gameplay. Next, “the observer” could be defined as esports fans who frequently watch esports events or streamers' esports gameplay but whose esports gameplay is not equaled by their viewing consumption. As the required hardware level is low for watching Twitch or YouTube gaming, hardware enthusiasm can be equally low. Lastly, “recreational gamer” refers to low gameplay frequency, low hardware enthusiasm, and low viewing consumption. The recreational gamers may be similar to traditional casual gamers, who invest less money in games and play in short sessions. Utilizing aforementioned clusters, this study seeks to answer the following research question:

**Research Question:** Will there be differences on the six determinants (i.e., HM, HB, PV, EE, SO, and FL) between the four proposed groups?

This exploration of esports consumer segmentation—based on gameplay experience—will help establish clusters that better capture the heterogeneity of esports consumers. Furthermore, this focus will help practitioners develop products, services, and marketing plans that best fit the unique characteristics and needs of esports consumers.

## Literature Review

### Esports Gameplay Consumer Experience

Consumers' prior experience has been considered a significant variable impacting current and future consumption behaviors (Fishbein and Ajzen, 2011). For consumers' prior experience, scholars have classified consumer types such as “hardcore” gamers or “casual” gamers to identify gaming consumers' segments (Juul, 2010; Billieux et al., 2015; Manero et al., 2016; Yee, 2019). The consumer type classifications included various elements. For instance, hardcore gamers were referred to as individuals who invest a significant amount of time and money in games as a lifestyle preference. On the other hand, casual players tend to invest less money in games and play in short sessions between different activities (Juul, 2010). Juul also found both hardcore and casual gamers enjoy a challenge in gameplay and play for a long time, but casual gamers divide a long time into multiple short sessions. Based on the hardcore and casual gamers, Yee (2019) indicated additional midcore gamers as people who regularly play video games but not as much as hardcore gamers concerning time investment or competitiveness. Based on gaming preferences of the genre (e.g., fighting, first-person shooting) and gameplay time, Manero et al. (2016) clustered gamers into four types: full gamers (individuals who play all kinds of games and invest very high frequency), hardcore gamers (individuals who mostly play first-person shooting, fighting, adventure, and sport gamers and invest above the general frequency), casual gamers (individuals who usually play music, social, adventure, and sport games and invest less than the general frequency), and non-gamers/occasional gamers (individuals who mostly play music and social games at a very low frequency). For problematic involvement in online gamers, Billieux et al. (2015) identified more specific subgroups: recreational gamers and social gamers from casual gamers as a non-problematic group and the escaper, the achiever, and hardcore gamers as a problematic group. For clustering, Billieux et al. (2015) used various elements such as impulsivity, self-esteem, gameplay time, sensation-seeking, and drive, to explore the problematic behaviors of gamers.

In addition, esports consumers are closely related to the hardware market because mobile technology has been growing dramatically as a gaming device rather than consoles or personal computers (PCs) as mobile esports games have gained popularity (Newzoo, 2019). For example, the gaming performance of esports mobile games has increased, and esports fans may upgrade their mobile phone to play esports games. In this sense, PC users also are likely to upgrade their PCs to participate in the latest hardware trends in order to play esports games with a high-quality setting. To define esports gameplay consumers, the need has escalated for evolving gameplay consumer type by diverse gameplay consumer experiences. In order to answer this need, more diverse segments of gameplay consumers have been proposed (Newzoo, 2019; Gamedesigning.org, 2020). Specifically, based on gameplay time, watching others gameplay, and gaming hardware, Gamedesigning.org (2020) indicated the six different gameplay consumers (hardcore gamer, casual gamer, the mobile gamer, the online gamer, the observer, and the armchair general). The mobile gamer, the online gamer, and the armchair general were divided based on the following gaming hardware: mobile phone, PC, and gaming console. The observer was defined as a gameplay consumer who is likely to watch other gameplay via live-streaming platforms such as Twitch and YouTube. Newzoo (2019) proposed the eight personas (e.g., ultimate gamer, all-around enthusiast, time filler). While diverse segments of esports consumers were proposed, the standards of clusters might not be consistent and systematic. For example, the segments of Gamedesigning.org (2020) might not catch the groups such as hardcore mobile gamers and casual mobile gamers. Newzoo's (2019) segmentation categorized four gameplay-related groups (ultimate gamer, all-around enthusiast, conventional player, and time filler) while categorized only two viewer-related groups (i.e., popcorn gamer and backseat viewer). Also, while the conventional player group included gameplay, hardware purchasing habit, and watching others' gameplay features, other groups include only one or two features.

Thus, this study proposes more consistent esports consumer clusters based on focal esports consumption, gameplay, viewing, and purchasing hardware enthusiasm. The esports consumers who have high hardware enthusiasm seem to have high esports gameplay intention. Considering gameplay consumers tend to upgrade their hardware for a better gameplay experience, hardware enthusiasm might be considered a peripheral gameplay consumption element. Like the free-to-play revenue model in esports games, accessibility to playing esports games has been considered as one of the most important factors. Thus, the required level of gaming hardware for popular esports games is most likely low so they can be played widely.

With the theoretical background, esports gameplay time, watching esports contents, and hardware enthusiasm might need to be considered to understand gameplay consumer segments deeper in the esports context. According to the Nielsen: Games 360 US Report 2018, the average gaming hours of esports consumers were ~7 h per week. In this study, the self-perception of consumption frequency was weighted more than actual hours because of the esports context's features. Specifically, some gameplay consumers might think they are hardcore gamers but cannot play as they used to because of other time-constraining responsibilities (Newzoo, 2019). Despite this, these consumers may still perceive themselves as being hardcore gamers regardless of total time spent consuming esports. Similarly, some gameplay consumers may display the characteristics and behaviors of casual gamers—usually using gameplay for social interaction purposes (Manero et al., 2016)—yet spend more than 7 h per week gaming, depending on their weekly allotment of free time. While their gameplay time may exceed the 7 h average, the consumers' behaviors and self-perception of gameplay consumption still may categorize the consumer as a casual gamer. Thus, this study explores consumers' gameplay behavior—in regard to the weekly amount of time spend gaming—within the scope of self-perception; study respondents were asked to self-reflect to determine if they perceived themselves as playing more or less than the average weekly amount of time spent gaming. Doing so accounts for consumers' fluctuating amount of available free time.

With regard to gaming hardware, 73% of esports consumers own a game console (e.g., Xbox), 43% have a handheld system for gaming (e.g., Nintendo Switch), 29% own a VR (virtual reality) device, and 25% have a mobile VR device (Entertainment Software Association, 2020). Considering some individuals have multiple gaming devices, owning the gaming devices may be closely related to the type of esports gameplay consumers' segmentations. Also, esports consumers might upgrade their gaming devices or purchasing a new type of hardware (e.g., VR) to play their favorite esports games with better visual or functional conditions (Newzoo, 2019; Gamedesigning.org, 2020). In this study, purchasing a game to start playing or purchasing an in-game item was not included. As there are many esports games (e.g., League of Legends, Fortnite, Dota2, VALORANT, etc.) using the free-to-play strategy, which is based on “Freemium” business models (Niemand et al., 2019), purchasing for starting of gameplay may not be applied to general purchasing consumptions. In the esports context, the in-game items are typically for boosting the convenience of gameplay or are cosmetic items. Those in-game items usually do not affect the gameplay performance, because pay-to-win system can be seriously harmful to fair competition based on players' skills or teamwork. Macey et al. (2020) found gameplay intention positively influences purchasing intention of in-game items. While it is unnecessary to buy cosmetic items for gameplay, consumers might want to buy those for their favorite characters or weapons when esports consumers play a lot. However, there is a lack of theoretical background when esports consumers start to commit and buy in-game items. For instance, some esports consumers spend a lot of time on gameplay but may not purchase the in-game times. On the other hand, the purpose of purchasing the gaming consoles and gears is only for gameplay. Thus, based on three esports experiences (gameplay, viewing, and hardware enthusiasm), the current study proposed four different types of esports gameplay consumers (Figure 1).

### The ESC Model

To measure esports gameplay intention, Jang and Byon (2020a) proposed the ESC model that includes six determinants (HM, HB, PV, EE, SO, and FL). The HM can be referred to as the pleasure of playing esports games. The factor of HB is the perception of future automatic behaviors regarding esports gameplay. The PV is the consumers' perception of how worthwhile it is to spend their money on esports gameplay. EE refers to difficulty level when learning to play esports games. The factor of SO is defined as the influences of friends or family regarding esports gameplay intention. Lastly, FL is defined as the absorbed status in the playing of esports game, so the real world might be forgotten. The authors found HM, PV, EE, and FL significantly impact esports gameplay intention.

In order to expand the utility of the ESC model, the relationships between the six determinants and esports gameplay intention have been examined under the boundary conditions of the esports genre (Jang and Byon, 2020b) and gender (Jang and Byon, in press). In order to further extend the ESC model's applicability and understand esports gameplay consumption better based on the uniqueness of the esports context, this study empirically identified the proposed four esports gameplay consumer types by esports experience.

## Materials and Methods

### Measures

The survey had four sections: screening questions, esports experience, the six determinants of esports gameplay intention, and demographics. As a screening question, the types of gameplay consumers (i.e., non-gamer, recreational gamer, the observer, conventional player, all-around gamer) were asked for brief explanations of their esports experience, and respondents who selected “non-gamer” were automatically directed to the end of the survey. For the esports experience section, the high or low frequency of esports gameplay, watching esports content (e.g., streamers' live streaming, esports events), and hardware enthusiasm were measured. Following Manero et al. (2016), we measured esports experience by asking the following questions: “I think that I play my favorite esports game _____ the general frequency.” (*Note*: The “general frequency” is the frequency of typical players' gameplay as perceived by the respondent.) “I think that I watch my favorite esports game content (e.g., streamers' live streaming, esports tournaments) ______ the general frequency. (*Note*. The “general frequency” is the frequency of typical players' gameplay as perceived by the respondent.) The respondents who selected “above” were considered high frequency, and those selecting “under” were categorized as low frequency. For hardware enthusiasm, two descriptions for high enthusiasm (“I am most likely a hardware enthusiast. I own a lot gaming hardware or keep up with the latest hardware trends for gaming, including PC and mobile.”) and low enthusiasm (“I like to play esports games or watch other play, but I do not want to seriously spend money on hardware, such as upgrading a PC, mobile, or gaming console.”) were provided as options. This study uses perception-based self-reports for the following reasons. First, means are heavily influenced by extreme scores. While the average esports gameplay hours were ~7 h per week (Nielsen, 2018), according to more than 700,000 hardcore gamers profiles, a minimum 1-week play time was 20 h (Baumann et al., 2018). The high gameplay hours might pull the mean up so that average hours per week might not be an exact standard of the general frequency. Thus, the general frequency of using esports might need bandwidth. While self-report questionnaires might have comparatively lower fidelity than objective measurement, the greater bandwidth is one of the merits of using self-reports (Gonyea, 2005). Second, social-desirability bias (SDB) may have less influence on the self-reported values regarding esports consumption because esports consumptions are more like private behaviors rather than behaviors in public. Although the validity of self-reported values is typically doubted because of SDB, Fisher and Katz (2000) indicated that the SDB component from self-reports is likely to be better predictors of private behaviors.

This study conducted the ESC model's scale for the six focal constructs with 20 items (Jang and Byon, 2020a). The items measured were as follows: HM (3 items), HB (4 items), PV (3 items), EE (4 items), SO (3 items), and FL (3 items). A seven-point Likert scale anchored with (1) *strongly disagree* and (7) *strongly agree* was used.

### Data Collection Procedure

The online survey was created in an online surveying platform, Qualtrics, and received institutional review board approval from the researchers' host university. Once approved, the survey link was posted on the Amazon Mechanical Turk (M-Turk) website. Participants met the selection criteria for this study if all of the following qualifications applied: primary residence in the United States, 18+ years of age, esports gameplay experience, esports spectatorship/digital content consumptions, and 99% approval rates with a minimum of 100 previously accepted responses. According to the findings of Peer et al. (2014) study, the data from individuals in M-Turk who have survey response acceptance rates of 99% or above with a minimum of 100 previously accepted responses showed reliable quality; thus, the selection criteria were established to elicit quality data. As the qualifying question, the types of gameplay (non-gamer, recreational gamer, the observer, conventional player, all-around gamer) were asked for brief explanations of their esports experience. The explanation of non-gamer includes no experience of esports gameplay or spectatorship consumption. The participants of this survey must have both gameplay and watching others' gameplay experiences. If participants completed the surveys, those were reviewed for speeders and incomplete responses. For non-speeder surveys and completed responses, each respondent was paid US $1 as an incentive.

### Participants

To collect samples for all of the four gameplay consumer segments, the quotas function was used. For each group (high gameplay and high viewing = all-around gamer, high gameplay and low viewing = conventional player, low gameplay and high viewing = the observer, low gameplay and low viewing = recreational gamer), 200 was set as quota limits. Initially, a total of 800 observations were collected. After data screening, 101 were dropped because of incomplete surveys. Thus, a total of 699 usable observations were retained (recreational gamer: *n* = 225, the observer: *n* = 127, conventional player: *n* = 170, all-around: *n* = 177).

Participants' demographic characteristics were collected. Specifically, the 23–38-year-old age group (*n* = 447, 63.9%) made up the majority of participants: (18–20-year-old age group, *n* = 52, 7.4%; 39–49-year-old age group, *n* = 141, 8.4%; 50 years or older, *n* = 59, 8.4%). Regarding gender identity, 446 participants identified as men (64%), and 250 participants identified as women (35.6%). Additionally, three participants identified as gender variant/gender non-conforming (0.4%). The majority of ethnicity was White (*n* = 538, 77%), 79 Asians (11.3%), and 56 Blacks or African Americans (8%). The largest household income was between US $40,000 and $69,999 (*n* = 220, 31.5%); 183 (26.2%) had $10,000–$39,999, and 153 (21.9%) had $70,000–$99,999.

According to Nielsen's report (2018), the US esports fans' demographics revealed 75% were men, 75% were aged 18–34 years, and the average household income was $58,900. Considering the sample's demographic in this study (63.7% % men; 71.3% were 18–38 years old; 31.5% had household income of $4,000–$69,999), this aligned with esports consumers' demographic characteristics.

### Data Analyses

This study aimed to examine differences in esports gameplay determinants between esports gameplay consumer segments by esports experience. First, psychometric properties of the measurement model were examined via confirmatory factor analysis (CFA). Second, a one-way multivariate analysis of variance (MANOVA) was then conducted to examine the significant differences across the four groups. Lastly, to identify distinct differences between group pairs, an analysis of variance (ANOVA) and multiple comparison tests were used for follow-up analyses. SPSS 25 and AMOS 25 were used to conduct the statistical analyses.

## Results

To examine the measurement model, the assumption test (outliers, normality, and multicollinearity) and the instrument's psychometric properties were tested. As the results of skewness (−1.20 to −0.19) and kurtosis (−0.35 to 2.43) were under the suggested criteria (Hair et al., 2010), there was no normality issue. Also, there were no outliers by the boxplot test. All of the values of the correlation were <0.85 (Kline, 2015), and variance inflation factor ranged from 1.774 to 3.353, which indicated that there was no multicollinearity issue. The model fit of a total sample was acceptable (χ^2^ = 662.15, *df* = 155, *p* < 0.05; χ^2^*/df* = 4.27; CFI =0.94; and RMSEA = 0.068). The range of factor loadings was from 0.75 to 0.88 (Table 1). Based on the factor loadings, validity and reliability were acceptable. The range of AVE values for variables was 0.61 to 0.76, and those were above the squared correlations between variables (Table 2). For the instrument's psychometric properties for each group, a CFA was examined (recreational gamer: *n* = 225, the observer: *n* = 127, conventional player: *n* = 170, all-around gamer: *n* = 177). Although the ideal sample size in CFA would be 200 (Kline, 2015), theoretically, a 100–150 sample size is also recommended for CFA as comparatively liberal rules of thumb (Hair et al., 2010).

**Table 1:** Indicator loadings (λ), construct reliability (CR), and average variance extracted (AVE) for the variables and items.

**Variables**	**λ**	**Total sample (*N* = 669)**	**Recreational gamer (*n =* 225)**	**The observer (*n =* 127)**	**Conventional player (*n =* 170)**	**All-around gamer (*n =* 177)**
***Hedonic motivation*** (CR/AVE)		0.84/0.63	0.85/0.66	0.73/0.48	0.86/0.67	0.85/0.65
Playing (my favorite esports game) provides me with a lot of enjoyment		0.86	0.88	0.80	0.91	0.82
I am pleased when I play (my favorite esports game)		0.78	0.80	0.71	0.77	0.80
I enjoyed playing (my favorite esports game) because it is exciting		0.75	0.75	0.55	0.77	0.80
***Habit*** (CR/AVE)		0.86/0.61	0.86/0.61	0.84/0.57	0.80/0.50	0.85/0.60
The playing of (my favorite esports game) has become a habit for me		0.79	0.76	0.78	0.78	0.78
Playing (my favorite esports game) has become automatic to me		0.81	0.82	0.83	0.64	0.82
If I have to select a task in my leisure time, it is an obvious choice for me to play (my favorite esports game)		0.79	0.78	0.77	0.69	0.79
Playing (my favorite esports game) has become natural to me		0.74	0.76	0.64	0.72	0.69
***Price value*** (CR/AVE)		0.86/0.67	0.86/0.67	0.88/0.71	0.88/0.71	0.78/0.55
Playing (my favorite esports game) is reasonably priced		0.76	0.74	0.84	0.80	0.67
Playing (my favorite esports game) is a good value for the money		0.82	0.85	0.85	0.83	0.72
At the current cost (my favorite esports game) provides a good value		0.87	0.86	0.84	0.90	0.82
***Effort expectancy*** (CR/AVE)		0.86/0.61	0.88/0.65	0.84/0.57	0.81/0.51	0.83/0.55
Learning how to play (my favorite esports game) is easy for me		0.83	0.86	0.78	0.77	0.79
My interaction with (my favorite esports game) is clear and understandable		0.66	0.70	0.56	0.59	0.64
I find (my favorite esports game) easy to play		0.81	0.83	0.81	0.77	0.75
It is easy for me to become skillful at playing (my favorite esports game)		0.80	0.82	0.84	0.72	0.77
***Social influence*** (CR/AVE)		0.88/0.71	0.85/0.66	0.87/0.70	0.90/0.75	0.86/0.68
People who are important to me think that I should play (my favorite esports game)		0.87	0.82	0.88	0.89	0.88
People who influence my behavior think that I should play (my favorite esports game)		0.81	0.78	0.86	0.84	0.72
People whose opinions that I value prefer that I play (my favorite esports game)		0.84	0.83	0.76	0.86	0.86
***Flow*** (CR/AVE)		0.91/0.76	0.92/0.80	0.90/0.75	0.84/0.64	0.89/0.73
I frequently experience flow when I play (my favorite esports game)		0.87	0.90	0.85	0.78	0.83
In general, I have frequently experienced flow when playing (my favorite esports game)		0.88	0.88	0.89	0.82	0.90
Most of the time, when I play (my favorite esports game), I feel I am experiencing flow		0.87	0.90	0.85	0.80	0.84

**Table 2:** Correlations among all variables.

**Total sample**	**AVE**	**1**	**2**	**3**	**4**	**5**	**6**
1. Hedonic motivation	0.63	1					
2. Habit	0.61	0.74^*^ (0.55)	1				
3. Price value	0.67	0.56^*^ (0.31)	0.51^*^ (0.26)	1			
4. Effort expectancy	0.61	0.63^*^ (0.39)	0.68^*^ (0.46)	0.51^*^ (0.26)	1		
5. Social influence	0.71	0.28^*^ (0.08)	0.49^*^ (0.24)	0.33^*^ (0.11)	0.47^*^ (0.22)	1	
6. Flow	0.76	0.66^*^ (0.43)	0.72^*^ (0.52)	0.50^*^ (0.25)	0.74^*^ (0.55)	0.54^*^ (0.29)	1
**Recreational gamer**	**AVE**	**1**	**2**	**3**	**4**	**5**	**6**
1. Hedonic motivation	0.66	1					
2. Habit	0.61	0.76^*^ (0.57)	1				
3. Price value	0.67	0.58^*^ (0.33)	0.53^*^ (0.28)	1			
4. Effort expectancy	0.65	0.61^*^ (0.37)	0.70^*^ (0.48)	0.59^*^ (0.35)	1		
5. Social influence	0.66	0.31^*^ (0.10)	0.45^*^ (0.20)	0.35^*^ (0.12)	0.41^*^ (0.17)	1	
6. Flow	0.80	0.61^*^ (0.37)	0.66^*^ (0.44)	0.48^*^ (0.23)	0.73^*^ (0.54)	0.54^*^ (0.29)	1
**The observer**	**AVE**	**1**	**2**	**3**	**4**	**5**	**6**
1. Hedonic motivation	0.48	1					
2. Habit	0.57	0.57^*^ (0.32)	1				
3. Price value	0.71	0.65^*^ (0.42)	0.48^*^ (0.23)	1			
4. Effort expectancy	0.57	0.64^*^ (0.41)	0.52^*^ (0.27)	0.36^*^ (0.13)	1		
5. Social influence	0.70	0.19^*^ (0.03)	0.54^*^ (0.29)	0.22^*^ (0.05)	0.55^*^ (0.30)	1	
6. Flow	0.75	0.68^*^ (0.46)	0.65^*^ (0.43)	0.61^*^ (0.37)	0.71^*^ (0.51)	0.50^*^ (0.25)	1
**Conventional player**	**AVE**	**1**	**2**	**3**	**4**	**5**	**6**
1. Hedonic motivation	0.67	1					
2. Habit	0.50	0.76^*^ (0.58)	1				
3. Price value	0.71	0.42^*^ (0.18)	0.36^*^ (0.13)	1			
4. Effort expectancy	0.51	0.49^*^ (0.24)	0.62^*^ (0.38)	0.43^*^ (0.19)	1		
5. Social influence	0.75	0.17^*^ (0.03)	0.40^*^ (0.16)	0.21^*^ (0.04)	0.36^*^ (0.13)	1	
6. Flow	0.64	0.62^*^ (0.38)	0.74^*^ (0.55)	0.28^*^ (0.08)	0.69^*^ (0.47)	0.48^*^ (0.23)	1
**All-around gamer**	**AVE**	**1**	**2**	**3**	**4**	**5**	**6**
1. Hedonic motivation	0.65	1					
2. Habit	0.60	0.80^*^ (0.65)	1				
3. Price value	0.55	0.53^*^ (0.28)	0.54^*^ (0.29)	1			
4. Effort expectancy	0.55	0.74^*^ (0.55)	0.62^*^ (0.38)	0.44^*^ (0.19)	1		
5. Social influence	0.68	0.25^*^ (0.06)	0.34^*^ (0.11)	0.35^*^ (0.12)	0.37^*^ (0.14)	1	
6. Flow	0.73	0.72^*^ (0.52)	0.75^*^ (0.56)	0.52^*^ (0.27)	0.67^*^ (0.45)	0.43^*^ (0.18)	1

* *p < 0.001*.

For the recreational gamer group, the model fit was acceptable (χ^2^ = 372.45, *df* = 155, *p* < 0.05; χ^2^*/df* = 2.40; CFI = 0.93; and RMSEA = 0.079). All of the values of factor loadings fell between 0.70 and 0.90 (Table 1), which were above the cutoff value of 0.50 (Hair et al., 2010). The variables and items showed proper discriminant validity and reliability. As evidence of discriminant validity, the values of AVE were above the squared correlations between variables (Table 2; Fornell and Larcker, 1981). For reliability, all variables' CR values exceeded the suggested threshold of 0.70 (Hair et al., 2010).

The model fit of conventional player group showed a good fit (χ^2^ = 273.58, *df* = 155, *p* < 0.05; χ^2^*/df* = 1.77; CFI = 0.92; RMSEA = 0.078). The range of factor loadings was 0.59–0.89, which exceed the threshold of 0.50 (Hair et al., 2010). Overall, the discriminant validity and reliability were acceptable (Table 1). However, the values of AVE for the HM were slightly lower (0.48) than the suggested threshold of 0.50 (Hair et al., 2010). That may have been due to a low factor loading for an item measuring HM that was relatively lower (0.55), while it exceeded the liberal cutoff value of 0.50, but not the stringent criterion of 0.70 (Hair et al., 2010). Despite this issue, we decided to keep the item because of its theoretical importance. Discriminant validity was ensured as the squared correlations were below AVE values (Table 2; Fornell and Larcker, 1981). The CR values also support reliability by exceeding the threshold (0.70) (Hair et al., 2010).

Conventional player group's model fit also showed a good fit (χ^2^ = 286.45, *df* = 155, *p* < 0.05; χ^2^*/df* = 1.85; CFI = 0.93; RMSEA = 0.071). The items' values of factor loadings (0.59–0.91) were above the cutoff value of 0.50 (Hair et al., 2010), and the constructs' values of AVE (0.50–0.75) were >0.50, which indicated convergent validity (Table 1). The discriminant validity (Table 2) and reliability (CR; 0.80–0.90) were also supported.

Lastly, the model fit of all-around gamer represented an acceptable fit (χ^2^ = 409.41, *df* = 155, *p* < 0.05; χ^2^*/df* = 2.64; CFI = 0.88; RMSEA = 0.097). The range of factor loadings (0.64–0.90) was above the cutoff value of 0.50 (Hair et al., 2010), and the AVE values (0.55–0.73) were above 0.50 (Table 1). The discriminant validity (Table 2) and reliability (CR; 0.80–0.90) were also supported. Also, the AVE values were above the squared correlations between variables (Table 2; Fornell and Larcker, 1981), evidencing discriminant validity. For reliability, the CR values were (0.78–0.89) above the threshold of 0.70. Overall, the four groups' factor analysis results show the instrument has a proper psychometric quality for conducting measurement invariance tests.

### Multivariate Analysis of Variance

A one-way MANOVA was conducted to examine the impact of the four groups. Box test was examined for the assumptions (i.e., multivariate normality and homogeneity of the covariance matrices) with the four groups (recreational gamer: *n* = 225, the observer: *n* = 127, conventional player: *n* = 170, all-around gamer: *n* = 177). As the Box test was significant, which indicated there are no equal covariance matrices, every 127 cases were randomly selected from recreational gamer (*n* = 225), conventional player (*n* = 170), and all-around gamer (*n* = 177) groups to make the same sample sizes (Field, 2013). As the MANOVA test statistics are robust to violations of the assumptions when the sample sizes are equal (Field, 2013), the four groups' equal sample sizes were used (recreational gamer: *n* = 127, the observer: *n* = 127, conventional player: *n* = 127, all-around gamer: *n* = 127). The six determinants were examined for their differences and similarities across the four groups. The results of Wilks Λ showed that significant differences exist across the means of four groups on a combination of outcome variables [Λ = 0.75, *F*_(60, 1, 447)_ = 2.39, *p* < 0.001].

### Univariate Tests

Table 3 presents the results of the multiple univariate tests and Tukey HSD (honestly significant difference) *post-hoc* tests of group pairs. The groups' equal variance and the means' normal distribution met the assumptions of using Tukey HSD to control family-wise error rate. An ANOVA showed the significant effects of the four groups on the six determinants of esports gameplay intention [HM: *F*_(3, 504)_ = 8.076, *p* = 0.000, partial η^2^ = 0.046; HB: *F*_(3, 504)_ = 19.413, *p* = 0.000, partial η^2^ = 0.104; PV: *F*_(3, 504)_ = 4.503, *p* = 0.004, partial η^2^ = 0.026; EE: *F*_(3, 504)_ = 13.142, *p* = 0.000, partial η^2^ = 0.073; SO: *F*_(3, 504)_ = 13.142, *p* = 0.000, partial η^2^ = 0.073; FL: *F*_(3, 504)_ = 14.502, *p* = 0.000, partial η^2^ = 0.079]. The results support the group differences across the four segments. The results of subsequent *post-hoc* comparisons indicated HB, EE, SO, and FL showed significant differences on the most group pairs. However, HM and PV showed statistical differences on the two or three pairs (Table 3). The differences and similarities of the six antecedents of esports gameplay intention contributed to answering the research question. The interpretation of the results is discussed in the following section.

**Table 3:** Means, univariate tests, and *post-hoc* tests by the four groups.

	**Recreational gamer (G1)**	**The observer (G2)**	**Conventional player (G3)**	**All-around gamer (G4)**	**Univariate tests**		**Tukey HSD** ***post-hoc*** **tests**	
	**Mean (SD)**	**Mean (SD)**	**Mean (SD)**	**Mean (SD)**	***F*** **(between-G, within-G** ***df*****)**	***p* (*η^2^*)**	**Groups**	***p***
HM	5.787 (0.95)	5.929 (0.70)	6.01 (0.83)	6.27 (0.82)	8.076 (3, 504)	0.000^*^ (0.046)	G1 vs. G2	0.497
							G1 vs. G3	0.136
							G1 vs. G4	0.000^*^
							G2 vs. G3	0.875
							G2 vs. G4	0.004^*^
							G3 vs. G4	0.044^*^
HB	5.193 (1.21)	5.510 (0.95)	5.701 (0.88)	6.091 (0.74)	19.413 (3, 504)	0.000^*^ (0.104)	G1 vs. G2	0.043^*^
							G1 vs. G3	0.000^*^
							G1 vs. G4	0.000^*^
							G2 vs. G3	0.388
							G2 vs. G4	0.000^*^
							G3 vs. G4	0.007^*^
PV	5.430 (1.06)	5.533 (1.06)	5.638 (1.05)	5.877 (0.88)	4.503 (3, 504)	0.004^*^ (0.026)	G1 vs. G2	0.853
							G1 vs. G3	0.364
							G1 vs. G4	0.003^*^
							G2 vs. G3	0.843
							G2 vs. G4	0.036^*^
							G3 vs. G4	0.241
EE	5.289 (1.06)	5.443 (0.95)	5.789 (0.80)	5.921 (0.94)	13.281 (3, 504)	0.000^*^ (0.073)	G1 vs. G2	0.535
							G1 vs. G3	0.000^*^
							G1 vs. G4	0.000^*^
							G2 vs. G3	0.013^*^
							G2 vs. G4	0.000^*^
							G3 vs. G4	0.656
SO	4.199 (1.28)	4.617 (1.14)	4.661 (1.25)	5.155 (1.19)	13.142 (3, 504)	0.000^*^ (0.073)	G1 vs. G2	0.033^*^
							G1 vs. G3	0.014^*^
							G1 vs. G4	0.000^*^
							G2 vs. G3	0.991
							G2 vs. G4	0.003^*^
							G3 vs. G4	0.007^*^
FL	5.123 (1.24)	5.299 (1.02)	5.669 (0.84)	5.874 (0.91)	14.502 (3, 504)	0.000^*^ (0.079)	G1 vs. G2	0.509
							G1 vs. G3	0.000^*^
							G1 vs. G4	0.000^*^
							G2 vs. G3	0.019^*^
							G2 vs. G4	0.000^*^
							G3 vs. G4	0.372

*HM, hedonic motivation; HB, habit; PV, price value; EE, effort expectancy; SO, social influence; FL, flow*.

* *Significant*.

## Discussion

This study aimed to identify adequate esports gameplay consumer groups in the context of esports based on their esports game experience. MANOVA and follow-up tests supported the empirical evidence of the four groups' differences by esports experience. Specifically, the esports gameplay consumer segments by esports experience were theoretically proposed and empirically supported. Thus, the esports consumers' clustering contributed to more efficient marketing, so targeted marketing may lead to effectively reaching the right people. After all, targeted marketing may allow for better investment returns. Specifically, HM and PV showed statistical differences on the pairs related to the all-around gamer. The differences with all-around gamer may be obvious because of the highest mean values of all-around gamer group. Excepting those differences, HM and PV showed non-statistical differences across the groups. The results indicated HM and PV may be considered as general factors that may work for overall esports consumers, which means all of the four groups. While the mean values for HM (mean = 5.79) and PV (mean = 5.43) were the lowest for the recreational gamer across the four groups, those are higher than other factors such as HB (mean = 5.19), EE (mean = 5.29), SO (mean = 4.20), and FL (mean = 5.12). Thus, practitioners need to seriously consider the two factors, HM and PV, for their marketing strategies for general esports gameplay consumers from recreational gamer to all-around gamer.

Contrarily, the findings indicated SO, HB, EE, and FL as considerations for tailored marketing strategies targeting esports consumer groups. Specifically, SO identifies significant differences between recreational gamers and the observer. For distinguished marketing strategies between the observer and conventional player, practitioners need to consider EE and FL. To make a different approach between the conventional player and all-around gamer, this study's findings suggest HB and SO. For example, while both conventional player (high gameplay frequency and low viewing frequency) and all-around gamer (high gameplay frequency and high viewing frequency) play esports games with high frequency, the difference of two groups can be viewing frequency. The significant differences regarding HB and SO might be caused by watching others' esports gameplay experience. As all-around gamers may watch esports content live streaming with high frequency, they may also regularly visit their favorite streamers' channel and socialize with other viewers. This particular all-around gamers' socializing behavior might continue and influence their gameplay consumption, such as playing the esports game together with other gamers who met on the live-streaming channel after watching the streamer's broadcasting.

### Theoretical Contributions

The findings of this study make important theoretical implications. First, the proposed four segmentations (recreational gamer, the observer, conventional player, all-around gamer) were based on behaviors and prior experience of esports consumers. The proposed four segmentations were distinguished from other gamer categorizations by focusing on the behavioral factors with three focal esports consumptions (esports gameplay, watching others' gameplay, purchasing for gameplay). While previous gamer categorizations contributed to market segmentations, there was the lack in explaining esports consumers' behaviors properly. For instance, while Gamedesigning.org (2020) proposed the six gamer categorizations (hardcore gamer, casual gamer, the mobile gamer, the online gamer, the observer, and the armchair general), there was difficulty in explaining overlapped individuals such as hardcore gamer who prefers to play online games. For another example, Manero et al. (2016) indicated that casual gamers usually play music, social, and sport games and spend less than the general frequency. As Manero et al. (2016) included game genre preference, it would be difficult to describe esports consumers who invest tremendous time to play sport games such as NBA 2K or FIFA. Lastly, in Newzoo's (2019) eight personas, the difference between conventional player and hardware enthusiast was vague because both groups prefer the latest hardware trends. While conventional player included less watching of others' gameplay with hardware preference, hardware enthusiast did not include anything about watching and just included hardware preference. The proposed four clusters in this study contribute to the esports literature by explaining esports consumers more holistically. The behavioral prior experience of esports consumers (esports gameplay, viewing esports content, and hardware enthusiasm) aligned with focal esports consumption based on the esports context's unique features (Jang and Byon, 2020a; Jang et al., 2020; Macey et al., 2020). As the proposed four clusters categorized esports consumers based on their behavioral prior experiences, we believe the clusters are considered more appropriate and benefit practitioners to explain and segment esports gameplay consumers.

Second, the findings of this study have contributed to the esports literature by exploring esports consumption experiences. Recently, esports consumer segmentation studies were conducted (Pizzo et al., 2018; Jang and Byon, 2020b, in press). Specifically, esports genre (Jang and Byon, 2020b) and gender (Jang and Byon, in press) were used as moderators between esports gameplay intention and its drivers. To predict an individual's intention to engage in a behavior, the Theory of Planned Behavior focused on behavioral control based on prior experience (Fishbein and Ajzen, 2011). As such, the importance of prior experience and its impact on consumers' behaviors are continuously found in various contexts (Venkatesh et al., 2012; Blut and Wang, 2019; Heiny et al., 2019). The esports prior experiences that were used for the proposed four segmentations might have potential to be served as moderator in esports consumer behavior studies in the future. Thus, in the context of esports, the suggested esports experience—supported by this study's findings—contributed to the growing body of knowledge in the esports literature.

### Practical Contributions

As for managerial implications, the findings of this study contributed to providing a more adequate reflection of esports consumers. While previous studies extended gamer clusters, the frequency of gameplay or game genre preference (Billieux et al., 2015; Manero et al., 2016; Yee, 2019) was reflected, but not esports content watching or hardware enthusiasm. In addition, although watching esports content via media or hardware enthusiasm was explored for esports consumer clusters (Newzoo, 2019; Gamedesigning.org, 2020), the consumer segmentations were too complex to measure because of uncertain criteria. The suggested esports experience (i.e., gameplay, viewing, and hardware enthusiasm) adequately reflects esports consumers as a whole and more accurately measures esports consumer clusters. Thus, this study's findings may help practitioners make data-driven decisions for consumer recruitment and retention efforts. For example, the self-perception of high or low gameplay frequency or viewing esports content or hardware enthusiasm may be effectively measurable. The suggested four gameplay consumer clusters (i.e., recreational gamer, the observer, conventional player, all-around gamer) more adequately reflect the unique characteristics of esports consumers' prior experience to better help practitioners target segment markets. For example, if gaming developer companies release a new esports game, practitioners may need to know what type of consumers like their newly released esports game. If the consumers are the observer type, practitioners may need to primarily focus on the connection of viewing based on the observer's features. The observer may prefer functions for watching other gameplay and briefly achieve their relatively low frequency of gameplay. The practitioners might want to have diverse strategies to approach different types of gameplay consumers. In practice, practitioners could use their understanding of the differences between groups to develop comprehensive training tutorials and challenge daily missions prompting more EE and FL. The two factors can make significant differences between conventional players and all-around gamers. Suppose the profile of primary consumers of a specific esports game has been changed from conventional players to all-around gamers. In that case, a better training system and challenging daily missions might be a helpful approach for all-around gamers by stimulating their EE and FL. If desiring to address another cluster, the practitioner's competency regarding understanding each segment's defining factors can better inform business practices.

### Limitations and Suggestions for Future Study

As with all research, this study has its limitations and direction for future research. First, this study considered esports gamer segments based on prior experience through drivers of esports gameplay intention. While this study contributed to identifying esports experience to adequately reflect esports consumers, this research model did not examine the extent to which the antecedents explain various esports consumptions for the four groups. Future esports research is needed to examine the relationship between the six antecedents and esports gameplay intention across the four groups.

Second, while this study provides empirical evidence for the four gameplay consumer types based on three consumption activities (i.e., esports gameplay, viewing esports content, and hardware enthusiasm), hardware enthusiasm might need to be more specifically conceptualized by considering potential elements such as in-game purchases. According to the findings of Macey and colleagues' study (2020), in-game purchases were also significantly related to other consumptions such as gameplay and viewing esports content. As such, esports consumers spend money on hardware and on in-game items. The consumption of in-game purchases is closely related to “Freemium” business models (Niemand et al., 2019), and the psychological background might be different to gaming hardware consumption. Future studies might need to consider in-game purchase consumption in the esports context to understand esports consumers' behavior in a more comprehensive perspective.

Third, as the aforementioned arguments in the literature review and methods state, this study utilized perception-based self-reports. While the measurement by self-reports on surveys completed by participants has been widely used in human–computer interaction research, the importance of examining actual behavior has been emphasized because of the response biases from the perception-based self-reports (Williams et al., 2017). Future studies may need to consider conceptualizing the general frequency of esports consumption more accurately. Based on the accurate general frequency, the groups may need to be clustered by more objective data such as consumption hours per week. Although the median split might be the solution, there are typical problems with median splits. For instance, if the median of esports gameplay hours was 9 h per week, 10 h may not significantly differ from 8 h. Also, 10 h may be significantly different from 30 h, even though those are supposed to be the same value. As one solution, some separation between the two groups can be created by deleting some responses around the median (Asada and Ko, 2016). If the proper general frequency can be identified, conducting K-means clustering may also be another solution to clustering similar data points and discover underlying patterns.

Lastly, this study did not use methods for discouraging speeders. Speeding in online survey can reduce the quality of responses and the accuracy of measurement. Future researchers may need to use methods such as interactive prompting technique. For example, this technique allows setting a minimal response time threshold. If respondents answered faster than the criteria, they receive a message encouraging their careful attention (Conrad et al., 2017).

## Data Availability Statement

The raw data supporting the conclusions of this article will be made available by the authors, without undue reservation.

## Ethics Statement

The studies involving human participants were reviewed and approved by Members of the Institutional Review Board at the University of West Georgia. The patients/participants provided their written informed consent to participate in this study.

## Author Contributions

WJ drafted the manuscript, collected the data, and performed data analysis. KB provided insight into conceiving the overall research idea, design of the study, execution of data analyses, and edits to the manuscript. JP contributed to writing a part of the introduction section and editing the manuscript. YT contributed immensely to the overall improvement in the quality of this study by giving critical feedback. All the authors participated and contributed to the research design.

## Conflict of Interest

The authors declare that the research was conducted in the absence of any commercial or financial relationships that could be construed as a potential conflict of interest.
